# Validation of a Method for Surveillance of Nanoparticles in Mussels Using Single-Particle Inductively Coupled Plasma-Mass Spectrometry

**DOI:** 10.1093/jaoacint/qsae024

**Published:** 2024-03-20

**Authors:** Are Bruvold, Stig Valdersnes, Katrin Loeschner, André Marcel Bienfait

**Affiliations:** Institute of Marine Research (IMR), PO Box 1870 Nordnes, N-5817 Bergen, Norway; University of Bergen, Department of Chemistry, PO Box 7803, N-5020 Bergen, Norway; Institute of Marine Research (IMR), PO Box 1870 Nordnes, N-5817 Bergen, Norway; University of Bergen, Department of Chemistry, PO Box 7803, N-5020 Bergen, Norway; Technical University of Denmark, National Food Institute, Kemitorvet 201, DK-2800 Kgs Lyngby, Denmark; Institute of Marine Research (IMR), PO Box 1870 Nordnes, N-5817 Bergen, Norway

## Abstract

**Background:**

Determining the concentration of nanoparticles (NPs) in marine organisms is important for evaluating their environmental impact and to assess potential food safety risks to human health.

**Objective:**

The current work aimed at developing an in-house method based on single-particle inductively coupled plasma-mass spectrometry (SP-ICP-MS) suitable for surveillance of NPs in mussels.

**Methods:**

A new low-cost and simple protease mixture was utilized for sample digestion, and novel open-source data processing was used, establishing detection limits on a statistical basis using false-positive and false-negative probabilities. The method was validated for 30 and 60 nm gold NPs spiked to mussels as a proxy for seafood.

**Results:**

Recoveries were 76–77% for particle mass concentration and 94–101% for particle number concentration. Intermediate precision was 8–9% for particle mass concentration and 7–8% for particle number concentration. The detection limit for size was 18 nm, for concentration 1.7 ng/g, and 4.2 × 10^5^ particles/g mussel tissue.

**Conclusion:**

The performance characteristics of the method were satisfactory compared with numeric Codex criteria. Further, the method was applied to titanium-, chromium- and copper-based particles in mussels.

**Highlights:**

The method demonstrates a new practical and cost-effective sample treatment, and streamlined, transparent, and reproducible data treatment for the routine surveillance of NPs in mussels.

Whereas organisms have evolved to coexist with natural nanoparticles (NPs) in the environment, the effects of exposure to anthropogenic incidental and engineered NPs are unknown (1). Identifying the number percentage of particles on the nanoscale has become a point of interest due to the unique properties of compounds on the nanoscale. This topic has only been studied rigorously in the last two decades (2), with scientists just recently having the tools to study and distinguish natural and anthropogenic NPs (3). For this reason, few studies have been performed on marine organisms (1) and coastal waters (4), less being known about NPs in the marine environment than any other principal earth compartment (2). This despite the ocean being a sink for contaminants and subject to, for example, deep sea mining and the disposal of mining waste containing NPs. Blue mussels are widely used bioindicators for monitoring anthropogenic pollution trends (5), capturing aggregated NPs in the marine environment (6) and having a lower biotransformation rate than fish, for example (5). They are further a popular food source; the European market is estimated at near to 600 000 tons per year (7).

Detecting and quantifying NPs in complex biological matrixes remain a challenge. However, single-particle inductively coupled plasma-mass spectrometry (SP-ICP-MS) has shown to be an invaluable tool holding further promise given instrumental improvements (8). Sample preparation for matrix degradation is generally required using alkaline, acidic, or enzymatic digestion prior to analysis (9). Stability of NPs and matrix effects may influence results requiring careful sample treatment and method development (8). NPs exhibit an extrinsic or media-dependent nature (10), demanding extra efforts toward standardization and validation (11). A number of extraction protocols have been employed (9), with enzymatic (mainly Proteinase K) and alkaline digestion (mainly tetramethylammonium hydroxide) generally showing the greatest promise for biological matrixes (12). Enzymatic digestion has been most extensively researched (13), while alkaline digestion has recently gained popularity due to its wide applicability (12) and has been found preferential for some applications (14), though inferior for others (15). However, alkaline digestion may be problematic for certain NPs such as Ag (16) due to the potential formation of NPs from ionic forms of the element. Further, tetramethylammonium hydroxide is avoided in many laboratories due to its hazards. Protamex^®^ (protease from *Bacillus* sp.) has previously been applied to the digestion of Atlantic salmon and yellowfin tuna (17, 18). Protamex is active in mild conditions at neutral pH and only slightly elevated temperatures, offering a quick, simple, robust, and safe alternative for matrix digestion, at a cost up to orders of magnitudes lower than for Proteinase K (19). This reduces the chances of the NPs undergoing transformations such as dissolution or agglomeration, and additionally provides applicability and practicability for routine use.

To collect data, conduct surveillance and official controls, it is a requirement to use validated methods with proven performance fit for the intended purpose (20). The Food and Agriculture Organization of the United Nations has stated that one of the most urgent challenges identified in relation to enforcing a regulatory framework is the lack of routine methods for NPs in food (21). Currently, relatively few NP types have been investigated and there are inconsistencies both in reported metrics and their determination (12). Furthermore, signal processing is often performed with untransparent commercial algorithms and inaccurate statistical models. Few validated methods have been published for NPs using ICP-MS [TiO_2_ in various matrixes (22, 23)] or SP-ICP-MS [Au and Ag in mammalian tissue and blood (24, 25), Ag in chicken meat (26, 27), Ag in confectionary (28), and TiO_2_ in crab sticks (29)]. To the best of our knowledge, no validated method exists for quantifying NPs in blue mussels (*Mytilus edulis*). A few in-house validated methods for other types of edible mussels exist. However, these generally do not assess intermediate precision (day-to-day variability) and overall uncertainty of the method. As no certified reference materials for NPs in food or biological tissue exist (12), spiking with NPs with known properties, ideally of the same type as the analyte, is required (30). While not generally considered a contaminant or a food safety issue, gold has been used as a model nanomaterial (31). Gold NPs are ideal in method development and validation studies for spiking since they are available with well-defined monodisperse size distributions. Further, gold has low environmental levels, high stability and sensitivity, and no abundant isobaric or polyatomic interferences using ICP-MS (32).

The objective of this study was to develop and validate a method for surveillance of NPs in blue mussels. The method was required to allow the cost-efficient analysis of larger sample sets for observing relative changes in particle concentrations of at least an order of magnitude, and over long time periods in relation to potential environmental and food safety issues, e.g., arising in connection to the initiation of mining activities and the dumping of mineral-containing mining waste. Reproducibility and control of false-positive and -negative rates were ensured by employing open-source signal processing developed in-house. By evaluating the method performance for particle diameter, particle number, and mass concentrations versus Codex Alimentarius general and numeric method performance criteria, it can be established whether the method is fit for the intended purpose (33). As a starting point for assessing the method performance, blue mussels were spiked with gold NPs of two sizes (30 and 60 nm) and analyzed on five different days. We assessed method quality parameters such as selectivity, LOD, working range, trueness, precision, and robustness for gold. The method was further applied to other types of NPs by analyzing three samples from different locations for the presence of NPs containing titanium (Ti), chromium (Cr), and copper (Cu). The analysis was repeated on 2 days, providing novel precision data on NPs in seafood. The method is intended to serve as a basis for subsequent method development toward surveillance of different NPs in marine samples.

## Experimental

### Sample Preparation

Samples of blue mussel (*Mytilus edulis*) were obtained from the regular program for monitoring of seafood at the Institute of Marine Research (IMR, Bergen, Norway; 34). An aggregate sample of 25 random blue mussels was prepared by following the IMR’s internal standard procedure for sample preparation of mussels for trace metals. First, the mussels were removed from the freezer and allowed to thaw overnight. The next day, the mussels were opened by cutting the sphincter muscle with a knife and the mussels were left standing upright for five minutes to allow any water inside the mussel to drain. The tissue inside was then removed with a blunt knife and transferred to a sieve for further collective drainage of water for five minutes following rinsing with ultrapure water (UPW). The aggregate sample was subsequently homogenized with a food processor and further homogenized by a high-speed homogenizer (Polytron Pt-2100, Kinematica AG, Switzerland) to increase homogeneity. Following homogenization, the test sample was transferred to polypropylene (PP) cups with screw caps for storage in the freezer until further sample preparation and analysis. Prior to analysis, a container of homogenized mussel sample was allowed to thaw overnight at 4°C. For the method validation, a blue mussel tissue test portion (1 g) was weighed into a PP tube. Method blanks were added 1 mL of UPW instead of mussel tissue. For test portions spiked prior to digestion, 100 µL of a gold NP dispersion of 30 or 60 nm size with a nominal concentration of 500 µg/L was added the mussel tissue or UPW blank, resulting in a nominal concentration of 50 ng/g particles/mussel tissue. For unspiked matrix blanks (only digested mussel tissue, no gold NP), 100 µL UPW was added instead. For the test portions to be spiked after digestion, nothing was added. Then, 3 mL enzyme solution was added to each tube prior to the PP tubes being placed horizontally on a Unimax incubator heat-shaker (Heidolph, Germany) at 300 revolutions per minute and 50°C for 1 h. Matrix blank test solutions were then split, and one parallel spiked appropriately followed by dilution with UPW to the final concentration corresponding to a 500 times dilution of the mussel tissue. For every dilution step, tubes were thoroughly mixed with a vortex-shaker (MS1 Minishaker, IKA, Germany). For the method applicability demonstration, the enzyme solution was diluted by an extra factor of 5 and allowed to incubate overnight. In addition, UPW added to the method blanks in place of mussel tissue was homogenized in the same way as the mussel tissue. To properly simulate the mechanical abrasion of the mussel tissue, blanks containing homogenized polyethylene beads were also investigated (*see*  Supplemental Figure 2). Another 12-fold dilution was performed for the method application to environmental samples to decrease the background, resulting in a total dilution factor of 6000.

### Measurement Standards and NPs

An ionic standard for gold was purchased from Spectrascan, Ski, NO (SS-1118N) and used for calibrating the ICP-MS and for determination of transport efficiency. Spherical gold NPs of 60 and 30 nm nominal diameters with nominal concentrations of 50 mg/L were bought from Perkin Elmer, Waltham, MA (N8142303, lot no. E2840N and N8142300, lot no. SPD543N, respectively) and used both as reference materials for the determination of transport efficiency and for the spiking experiment. Ionic standards were stored at room temperature and NP standards were stored in a refrigerator at 4°C for approximately 18 months. The reference samples for the ACEnano proficiency test (PTs) were used to determine trueness as a z-score for Au NPs in UPW (Institute of Food Safety (RIKILT) 2018–02; 35). For the method demonstration on NPs of Ti, Cr, and Cu, ICP standards of ionic Ti from Spectrascan (SS-1164), Cr from Fluka Analytical (68131), and Cu from Sigma-Aldrich (68921) were used. NanoXact 60 nm Gold 50 mg/L, lot TJC0086, was used for calibration, and TiO_2_ NPs with a primary particle size of 115 nm from the Joint Research Centre ’s Nanomaterial Repository (JRCNM10200a, ID: 010118) and Cu(II) oxide nanopowder from Sigma-Aldrich (544868) were used to estimate peak widths or recovery.

### Reagents

UPW with a resistivity of 18.2 MΩ·cm at 25°C (Elix Progard TNP and Milli-Q Advantage A10, Merck Millipore, MA, USA) was used for aqueous dilutions. Nitric acid (65% EMSURE for analysis, Merck, Darmstadt, Germany) was used for standards and rinsing. Protamex (Merck), protease from *Bacillius* sp. was purchased from Sigma Aldrich, St. Louis, MO. A 200 g/L enzyme solution was prepared in UPW. Ionic and gold NPs used as standards and for spiking were prepared by dilution of the stock suspension in UPW. Final concentrations for gold NPs were 100 ng/L and 150 ng/L for reference materials and 500 µg/L for spiking. Ionic gold standards were prepared to concentrations of 0.5, 1 and 5 µg/L. Ionic standards for Ti, Cr, and Cu were prepared in 0.1% HNO_3_ to the same concentrations as the gold standards.

### Data Acquisition, Processing, and Visualization

Aliquots were analyzed using an Agilent 7900 ICP-MS fitted with a peristaltic pump and SPS-4 autosampler (Agilent Technologies, Santa Clara, CA, USA). Acquisition times of 2 and 3 min were used for the validation and method applicability demonstration, with nominal injection rate of 0.346 mL/min. Additional instrumental parameters are given in Supplemental Tables 6 and 7.

All data processing, calculations, and visualization was carried out in R version 4.2.1, while graphical elements were generated or edited using Affinity Designer 1.10.5, Vectormagic by Cedar Lake Ventures (Excelsior, MN, USA), and DALL-E 2 (OpenAI, San Francisco, CA, USA). For discriminating particle signals from background noise, a maximum peak intensity threshold was determined. This threshold was calculated based on the assumption that the background noise followed a Poisson distribution, with a constraint of a 95% probability of observing no more than one false positive per minute. This was done using the inverse Poisson cumulative density function in R using the local filtered rolling mean as the Poisson mean and a rate parameter of 1−0.05/600 000. Peaks above this threshold were integrated, subtracting the background corresponding to a rolling median with a correction for low-count backgrounds. For the multielement comparison, the lowest mass-normalized intensity threshold was used for all measurements to ensure similar false-negative rates. Transport efficiency was calculated in accordance with the particle size method (36). More specifically, a kernel density mode of the signal distribution resulting from the 60 nm Au NP standards was utilized to establish the signal per analyte mass. The mass flow rate at the detector was calculated from ionic standards and used in combination with the intake rate to establish the transport efficiency. Particle diameters were calculated assuming a spherical shape and a composition of pure gold with density 19.32 g/cm^3^, TiO_2_ with density 4.17 g/cm^3^, CuO with density of 6.31 g/cm^3^ and Cr_2_O_3_ with density of 5.22 g/${\text{cm}}_{.}^{3}$ Code and raw data is available through an online repository (37).

### Validation Setup and Calculation of Validation Results

**Figure 1.**
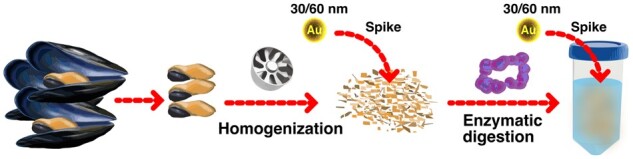
Schematic illustration of the sample treatment used in the validation study, spiking with gold NPs with nominal sizes of 30 or 60 nm.

Validation was carried out based on general in-house method validation guidelines from Eurachem and Nordic-Baltic Committee on Food Analysis (NMKL) (38, 39). Numeric values obtained for different validation parameters were compared to Codex Alimentarius numeric method criteria (33). The method parameters evaluated were particle mean diameter, particle mass concentration, and particle number concentration. The daily validation sequence was set up with ionic standards at the end to minimize contamination and memory effects. No outliers were removed. Data is shown in Supplemental Tables 1–3. An overview of the treatment is presented in Figure 1.

Selectivity was evaluated by assessing interferences in UPW versus the blue mussel matrix. This was achieved by comparing the parameters under investigation in test solutions both spiked and unspiked.

The working range of the method in terms of particle mass and number concentration was determined by analyzing ionic concentrations of gold and by approximating the probability of particle coincidence from Gaussian and Poisson statistics, respectively. For the mass or size per particle detection limit, a triangular peak shape was assumed using an estimated peak width of 500 µs for gold and 600 µs for the other elements, and a height corresponding to the peak intensity threshold. The mass per particle detection threshold could then be approximated by relating the signal area to the mass per signal from the calibration. For the method applicability demonstration, the highest mass-normalized threshold was utilized across all days and measurements to ensure an unbiased comparison with similar false-negative rates. For determination of the particle mass concentration detection limit, the mean pooled value of the mass concentrations of all blanks per day plus 3 times the standard deviation was used. The detection limit for the particle number concentration was similarly determined by a 99.7% Poisson confidence interval from the mean pooled values of procedural blanks on each day. The upper range of particle mass/size was not experimentally assessed.

Trueness of size as well as particle mass and number concentration were evaluated by recovery experiments and analyzing a PT. Recovery was assessed by comparing the measured values of gold NPs spiked in mussel tissues and NPs spiked in UPW to reveal matrix-specific bias, whereas laboratory bias was investigated by analyzing a PT sample consisting of gold NPs in UPW and calculating z-scores (*see* the equations in Supplemental Information). For laboratory bias, particle mass concentration was not assessed.

Precision as repeatability and intermediate precision were determined from replicates on each day over the 5 days using one-way analysis of variance as described by EURACHEM (39)

To account for random and systematic errors, measurement uncertainty was estimated for each measurand using a type B bottom-up approach. This was achieved through a Monte Carlo simulation using R version 4.2.1 and the metRology package (40) and is detailed in Supplemental Information. A coverage factor of 2 was used to calculate the expanded measurement uncertainty corresponding to a 95% confidence interval.

The stability of NP standards and spiked mussels was investigated by analyzing spiked method blanks and spiked mussels. Robustness was evaluated by using different batches of reagents and solutions on different days. Different personnel were also involved on different days during the five separate days of this validation study.

## Results and Discussion

### Selectivity

Selectivity as determined by evaluating instrument blanks and matrix blanks showed low concentrations in the unspiked aliquots as displayed in Table 1. The particles found were attributed to carryover and contributed to a higher detection limit, and could be further reduced by prolonging the rinsing procedure or changing its composition. This is consistent with the absence of isobaric interferences for particle signals at ^197^Au, with the only reported polyatomic interference being ^181^Ta^16^O (41). Studies have reported that the morphology of particles can impact the shape of their peaks (42). However, the current method lacks the ability to differentiate between gold NPs of various forms and size is determined based on assumptions regarding shape and density. It is possible to differentiate ionic from particulate species; however, it has been speculated that ionic species that are adsorbed onto particles could be falsely detected as particles (43). For other elements, interferents may be more abundant, yet interferents not causing particle signals will only contribute to a higher background and thus higher mass/size detection limits.

**Table 1.** Mean values and their corresponding standard deviations for each parameter in instrument and matrix blanks for all days, normalized to per kg mussel tissue

	Particle diameter, nm	Particle mass concentration, ng/g	Particle number concentration, #/g
Instrument blank (UPW)			
Unspiked	28.4 ± 5.6	0.1 ± 0.1	1.7 × 10^5^ ± 1.4 × 10^5^
Spiked with 30 nm gold NPs	35.7 ± 0.1	34.4 ± 1.3	7.1 × 10^7^ ± 3.0 × 10^6^
Spiked with 60 nm gold NPs	59.1 ± 1.0	23.2 ± 2.0	1.0 × 10^7^ ± 7.1 × 10^5^
Matrix blank (enzymatically digested mussel tissue)			
Unspiked	30.1 ± 3.1	0.8 ± 1.3	4.9 × 10^5^ ± 4.3 × 10^5^
Spiked with 30 nm gold NPs	32.8 ± 0.2	28.8 ± 2.8	7.4 × 10^7^ ± 6.9 × 10^6^
Spiked with 60 nm gold NPs	54.2 ± 1.1	18.7 ± 2.0	1.0 × 10^7^ ± 1.2 × 10^6^

**Figure 2.**
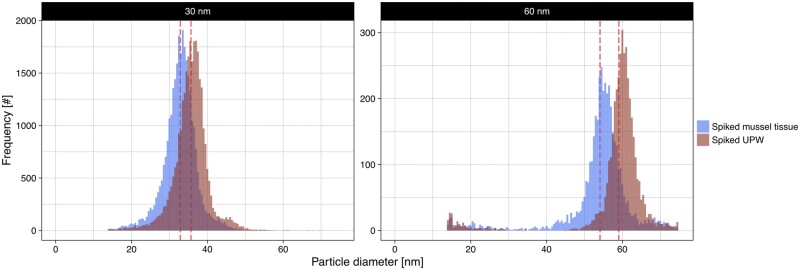
Size distributions of 30 nm (left) and 60 nm gold NPs (right) spiked into enzymatically digested blue mussel tissue (matrix blank) and UPW (instrument blank), illustrating the signal suppression by the blue mussel matrix. The dashed line indicates the pooled mean diameters across all days.

Matrix effects were evaluated by comparing NPs spiked to enzymatically digested mussel matrix (matrix blank) with NPs suspended in UPW (instrument blank). Mussel matrix resulted in an 8% decrease in mean diameter for 30 and 60 nm particles and a 19 and 21% decrease in terms of particle mass concentration in comparison to UPW (Figure 2; Table 1). For the particle number concentration, no significant difference could be found as determined by a *t*-test. From this, the particles appear stable in mussel tissue, as no aggregation or change in the size distribution can be observed. However, the blue mussel matrix was linked with a signal suppression resulting in lower size and mass concentration, yet did not have an impact on either the transport efficiency or the particle number concentration. Consequently, bias may be observed for particle diameters and particle mass concentrations depending on the matrix concentration and element measured. However, either by matrix-matching particle standards, or diluting the matrix, this difference could be mitigated or corrected for as discussed elsewhere (16, 44–46). However, correction was not performed due to it being unfeasible for other elements for which no reference materials are available, and as the method performance was fit for purpose and acceptable versus Codex criteria. For 60 nm particles a signal artifact or tailing effect can be observed appearing as a second population around 20 nm (Figure 2), occurring irrespective of matrix. Investigations of this phenomenon, inspecting both individual peak shapes and signal distributions from monodisperse gold NPs of eight different sizes in UPW, found a clear dependence on particle size (*see*  Supplemental Figure 3). Evidence signifies that this artifact is not an effect of the background or the signal processing, and may be a source for size bias and nonlinear particle signal to particle mass response for larger particles using SP-ICP-MS (see Supplemental Figure 4). This could lead to an overestimation in particle number concentration and an underestimation of particle sizes, whereas the mass concentration would remain the same. This artifact has also been described for Ag NPs in fruit juice (47) and UPW (48), the latter attributing this to the expansion of the ionic cloud as detailed in earlier works using microdroplet generators (49). However, the cause remains unclear. The artifact may often be eliminated as many vendors’ software relies on subjective manual adjustments for particle discrimination, and be dependent on instrumental setup and parameters. Widening of the particle size distribution using high nebulizer gas flow and sampling depth (50) may be related and underlines the importance of parameter optimization and reporting. This effect could explain seemingly contradictory reports of quantitative transfer for up to 1 µm SiO_2_ particles versus substantial nonlinear relationships at several hundred nm (51), and as low as around 100 nm (52, 53).

### Working Range

The working range for particle size or mass, particle mass concentration, and particle number concentration is limited at the lower end by the LOD, and in the upper range by a nonlinear response of the measured quantity.

The size or mass detection limit per particle corresponds to a Currie critical limit with a false-positive probability (α) of 8.33 × 10^−6^% per dwell under the assumption of Poisson noise, as detailed previously. This resulted on each day in a size of 18 nm or particle masses between 55 and 63 ag with a false-negative probability of 50%.

The upper detection limit per particle was not experimentally assessed in the validation setup. However, for larger particles, the aforementioned particle signal artifact, decreased transmission efficiency (54), detector saturation, and decreased transport efficiency can cause a bias. We therefore expect the upper limit of our method to be at least 100 nm for gold NPs, in line with previous reports (8, 50, 52, 53, 55) and separate experiments shown in Supplemental Figure 4. Above the working range, bias may be substantial. For monitoring nanomaterials, for example, in food for legislative purposes, NPs below 100 nm are of particular relevance (56). Non-detection of particles below the detection limit may be consequential since they may be numerous. In terms of particle mass concentration and particle number concentration, detection limits were determined to 1.7 ng/g mussel tissue and 4.2 × 10^5^ particles/g mussel tissue, respectively.

The upper working range for determination of particle mass concentration may be assumed to be equivalent to the working range for ionic forms of gold. Generally for ICP-MS, linear range spans many orders of magnitude (57), and for the current work it was found to be linear at least in the range of 0.25 to 5 ng/g: ionic standards used to determine transport efficiency were linear in this concentration range with *R*^2^ always 0.99997 or better (*see*  Supplemental Table 4). The upper working range for particle number concentration was defined as 5% particle coincidence and approximated from peak widths of 500 µs under the assumption of inter-arrival times following an exponential distribution and occurrence with time following a Poisson distribution. This resulted in a working range of up to 7.76 × 10^7^ particles/g mussel tissue for 30 nm and 5.96 × 10^7^ particles/g for 60 nm for the employed dilution factor of 500. This is predominantly in the range of reported environmental concentrations of metallic NPs or higher. For this reason, the working range is sufficient for determining expected environmentally relevant concentrations. If necessary, the method working range could be extended by increasing or decreasing the dilution factor. Transport efficiency was in the range 5.4–6.4%, corresponding to values reported with comparable setup (58–60).

### Trueness as Recovery

Trueness as recovery was evaluated by comparison of gold NPs in UPW with gold NPs spiked to method blanks (enzymatic digestion in the absence of mussel tissue) and mussel tissue to assess the matrix-specific bias. Mussel tissue was spiked both before (referred to as “blue mussel”) and after enzymatic digestion (referred to as “matrix blank”). As shown in Table 2, mean particle diameters were comparable between gold NPs spiked to mussel tissue before and after enzymatic digestion and the method blank with recoveries in the range of 86–92% for 60 nm particles and 92–93% for 30 nm particles. The previously described signal suppression (smaller diameters in comparison to gold NPs in UPW) may be caused by the enzyme as it is also observed in the method blank. It is also reflected by mussel tissue showing particle mass concentration recoveries at 77 and 84% for 60 nm and 76 and 81% for 30 nm, spiked prior to and after digestion, respectively. Corresponding particle number concentration recoveries were 94, 103, 101, and 103%. Thus, recoveries were similar whether spiked to the matrix before or after digestion or in UPW. This indicates that particles in the mussel matrix were stable during incubation and resulted in no change in transport efficiency.

**Table 2.** Recoveries of particle diameters, mass, and number concentrations in mussel tissue and method blanks versus UPW

Sample	Particle diameter, %	Particle mass concentration, %	Particle number concentration, %
30 nm gold NPs spiked to			
Blue mussel	92	77	94
Matrix blank	92	84	103
Method blank	93	17	20
60 nm gold NPs spiked to			
Blue mussel	90	76	101
Matrix blank	92	81	103
Method blank	86	13	18

Recoveries were within the Codex Alimentarius numeric method criteria interval of 40–120% (33). Analytical recoveries for enzymatic digestion of bivalves have been reported at 109%, 92%, and 85% for Ag (15, 61) and 95% for Ti (62) in terms of number concentrations, and 104% and 93% (15, 63) for mass concentrations, whereas recoveries for enzymatic digestion in general have been reported as low as near 20% due to incomplete digestion or high detection limit (12).

Trueness may also be calculated from the nominal sizes and concentrations of the reference material used for spiking, presented in Supplemental Table 5. However, the availability of certified reference materials is sparse and there is inherent uncertainty in the reference values, which may also change over time due to surface adsorption, dissolution, and aggregation. In the present work, recoveries versus nominal gold mass concentrations were found as low as approximately 40%. The low concentration recoveries were attributed to the stability of the reference material used, as further laboratory experience with the method using other NP standards consistently produced recoveries closer to 100%. Nonetheless, while trueness is crucial for comparison with, for example, regulatory limits, meaningful interpretations of trends and changes of environmental and food safety relevance can be derived from precision trends. It is worth noting that partly due to these limitations in the establishment of trueness, SP-ICP-MS is characterized as a screening technique and that unequivocal confirmation will have to be done by other techniques such as transmission or scanning electron microscopy (64). Observed recoveries were considered satisfactory for the purpose. However, spiked engineered NPs may behave differently than NPs found in the environment.

### Trueness from Proficiency Test

Trueness as a z-score was evaluated by analyzing the ACEnano PT samples in parallel for each experimental run to evaluate the laboratory bias (35). Relative trueness of particle number concentration and mean size was determined to be 112 and 94%, respectively, corresponding to satisfactory z-scores of 0.3 and 0.6. However, it should be noted that this PT was an ideal sample without complex matrix since there is also a lack of available PT in matrixes.

### Repeatability and Intermediate Precision

Repeatability and intermediate precision for size determination is generally more robust than concentration parameters due to the former being the mass equivalent diameter with a cubic dependence. This is reflected in the precision parameters obtained of below 2% (Table 3). For particles of different elements in various matrixes, e.g., gold and silver NPs in fruit juices (47), silver NPs in chicken meat (65), silver NPs in confectionery (28), TiO_2_ NPs in human urine (66), and gold NPs in UPW (67), repeatability standard deviations for size have mostly been reported below 10% and intermediate precision below 15%. Hence, the present method compares favorably to previous studies.

**Table 3.** Repeatability and intermediate precision for the particle diameter, particle mass concentration, and particle number concentration in terms of RSD, %

	RSD_repeatability_, %	RSD_intermediate precision_, %
Mean size		
Blue mussel + 30 nm	1.2	1.6
Blue mussel + 60 nm	1.1	1.4
Particle mass concentration		
Blue mussel + 30 nm	3.4	7.9
Blue mussel + 60 nm	5.0	8.8
Particle number concentration		
Blue mussel + 30 nm	2.1	7.1
Blue mussel + 60 nm	3.4	7.9

The precision of particle mass and number concentrations is also acceptable when compared to numeric criteria in Codex Alimentarius (33), with repeatability and intermediate precision up to 5% and 9%, respectively. These are lower in comparison to precision parameters reported for biological matrixes, e.g., with a repeatability of 8% for number concentration in mussels (61), a repeatability of 14% and reproducibility of 16% for mass concentration in urine (66), repeatabilities of 16–29% for number and mass concentrations in confectionary (28), and repeatability and reproducibility in confectionary and pristine solutions for number concentrations of 9–21% and 8–97%, respectively (59). Interlaboratory studies have reported repeatabilities between 7 and 18% and reproducibilities between 70 and 90% (8).

### Measurement Uncertainty

Although most uncertainties in SP-ICP-MS measurements are top-down estimates using the standard deviation of replicates or from precision parameters, some recent efforts have been made to more rigorously quantify their underlying sources (28, 68). Here, measurement uncertainties were determined by a conservative bottom-up Monte Carlo approach as detailed in the Supplemental Information along with quantity inputs. The expanded uncertainties using a coverage factor of 2 were determined to 8.8% for mean particle diameter, 37% for particle mass concentration, and 45% for particle number concentrations. While dependent on particle sizes, polydispersity, and calculation of uncertainty, these values fall in similar range as reported in the aforementioned studies. The greater uncertainty for particle mass and number concentration is primarily due to the uncertainty in the counting statistics at the lower end of the working range. Particle number concentrations are additionally dependent on both ionic and particulate reference materials. The uncertainty can be decreased by replicate measurements or measuring a larger number of particles, e.g., for 1000 measured particles, uncertainties decrease to 8.7, 16, and 29%, for size, mass, and number concentrations, respectively.

### Stability

Particle stability was assessed by the difference in particle number and mass concentrations in spiked method blanks versus matrix blanks spiked before and after digestion. As shown in Table 2, recoveries were highest for mussels spiked after digestion, followed by mussels spiked before digestion, and spiked method blanks. Method blanks deviated from the other matrixes with recoveries in the range of only 13–20% in terms of particle mass and number concentrations for 30 and 60 nm NPs. Matrixes of UPW and mussel matrix spiked before and after were found to be similar for particle number concentration recoveries, whereas particle mass concentration differences were attributed to matrix effects as discussed in *Trueness as Recovery*. These findings imply that the particles were stable in UPW and in mussel matrixes, with high recoveries and no apparent aggregation, but not when incubated with the protease mixture in isolation. Due to no change in the particle sizes measured, it is reasonable to assume surface adsorption to be the main mechanism, which has been reported as a potential source of bias in dilute particle suspensions (45, 69). This has implications for the selection of blanks and demonstrates that caution should be exercised when subtracting the blank and establishing detection limits based on blanks with different matrixes. The stability of spiked solutions has been observed to be poor; however, matrixes such as mussel tissue have a stabilizing effect.

### Method Application to Other NPs in Mussels

**Figure 3.**
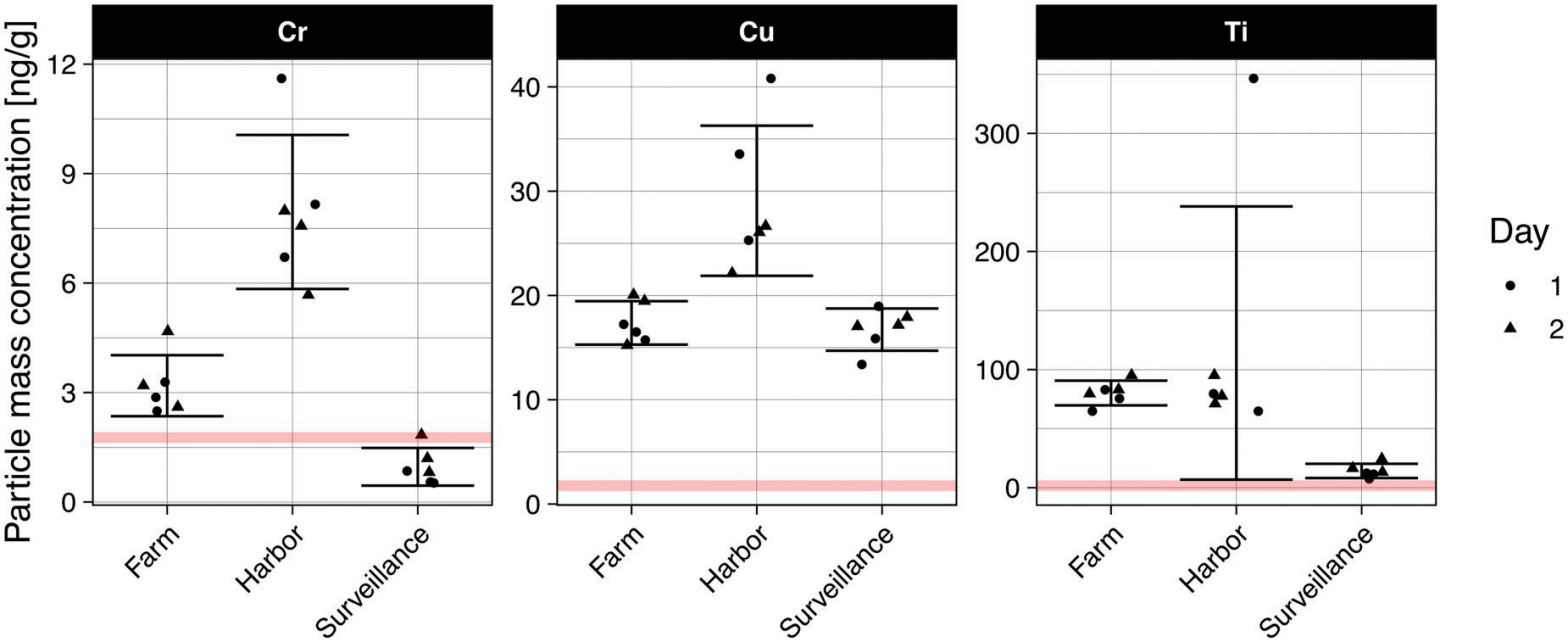
Particle mass concentrations in mussel tissue, the red line denoting the detection limit and error bars indicating the 95% confidence interval of the mean of six replicates over 2 days.

The application of the method to environmentally relevant particles of Cr-, Cu-, and Ti-containing particles was demonstrated using aggregate samples from three different locations, analyzed on 2 days. The aggregate samples represented a mussel farm, a harbor in central Bergen, and an unspecified location in the surveillance program. As demonstrated in Figure 3, the urban location, the harbor, exhibited significantly higher mass concentrations of Cr and Cu and had higher concentrations of Ti particles in comparison to the surveillance program sample. Particle number concentrations exhibited similar trends, shown in Supplemental Figure 1. Concentrations found for metal NPs can be orders of magnitude lower than for total metals (46, 70, 71), for which there are presently no regulatory limits. The between-days variability was in many cases smaller than the within-day variability, as found in other studies (28), indicating the method's robustness. The larger variance for the harbor sample may be attributed to sample heterogeneity due to differences in physiology. The RSDs were generally higher than observed for gold, yet acceptable as per the Codex criteria except for particle mass concentrations for Ti and Cr in some samples (Table 4). This is to be expected due to the added uncertainty of sampling in combination with the polydispersity of environmental particles for which a single, large particle may account for a substantial part of the total mass concentration. This was observed for Ti in one parallel sample from the harbor (Figure 2). Similarly, detection limits in terms of size are higher due to the presence of dissolved and interfering species causing elevated background noise. For ^48^Ti, spectral interference from ^48^Ca is possible, although a natural abundance of below 0.2% for this isotope limits this to a minor effect. However, the primary limitation of particle measurements of environmental samples is determining the trueness. Due to the absence of representative certified reference materials and PTs, both pure and in matrix, and the difficulty of determining environmentally relevant concentrations using alternative techniques, trueness generally cannot be established. However, mass recoveries based on spiking TiO_2_ NPs into mussels prior to incubation resulted in recoveries of 81–93% in comparison to a freshly prepared UPW solution (*see*  Supplemental Table 10). A second limitation is that determining the composition of particles is not achievable, although time-of-flight instrumentation can offer more information. However, SP-ICP-MS may serve as a fit-for-purpose standard method provided full validation is performed in a collaborative study. As environmental particle concentrations may increase logarithmically with decreasing size (72), comparisons on a particle number basis in particular are generally unsubstantiated unless detection limits are similar in terms of both false negatives and false positives. However, ensuring consistent sample and data treatment, different samples may be compared on a quantitative basis. This provides valuable insights into relative concentrations and allows surveillance of concentration of NPs in the marine environment.

**Table 4.** Precision parameters and detection limits across the 2 days for mass concentration in ng/g mussel tissue

	Mean, ng/g	Detection limit, ng/g	RSD_Repeatability_	RSD_Intermediate precision_
Ti				
Farm	80	3.4	11%	13%
Harbor	122	3.4	92%	90%^a^
Surveillance	14	3.4	31%	46%
Cr				
Farm	3	1.5	25%	25%^a^
Harbor	8	1.5	25%	26%
Surveillance	1	1.5	40%	57%
Cu				
Farm	17	0.4	11%	12%
Harbor	29	0.4	20%	26%
Surveillance	17	0.4	12%	12%^a^

a Total RSD is presented due to the variance being lower between days than within days.

## Conclusions

The study validated a method for determination of the size, mass, and number concentration of NPs in mussels using a cost-effective enzyme mixture suitable for routine applications. The method was highly selective for gold, although interference as signal suppression may cause an underestimation unless corrected for. The working range in terms of particle size ranged from 18 nm to at least 100 nm. The working range of particle number and mass concentration was from 1.7 ng/g to at least 5 ng/g and from 10^8^ to above 10^11^, respectively. Trueness as determined from PT samples without matrix and in matrix versus UPW was between 76 and 112% for all parameters. Repeatability and intermediate precision were below 2% for size and at most 9% for mass and number concentrations. Measurement uncertainties were 9, 16, and 29% for size, mass, and number concentrations, respectively. Thus, the overall method performance is acceptable in comparison to Codex recommendations and working ranges mostly span expected environmental levels and could be extended by dilution. As such, the method is fit for purpose in determining metal containing NPs such as gold in seafood samples with a similar matrix composition as mussels. While, as demonstrated, the high-precision data are suitable to monitor environmental trends, a remaining challenge is the difficulty of ascertaining the trueness, especially given the unavailability of certified reference materials and PTs for metal-containing NPs. However, by employing similar sample treatment, analysis, and detection limits through future method standardization, comparable quantitative data may be obtained for surveillance and monitoring purposes.

## CRediT Author Statement

**AB:** Conceptualization; Data curation; Formal analysis; Investigation; Methodology; Software; Validation; Visualization; Writing—original draft; Writing—review & editing. **SV:** Conceptualization; Data curation; Formal analysis; Funding acquisition; Investigation; Methodology; Project administration; Supervision; Validation; Writing—original draft; Writing—review & editing. **KL:** Conceptualization; Funding acquisition; Methodology; Supervision; Validation; Writing—original draft; Writing—review & editing. **AMB:** Conceptualization; Data curation; Formal analysis; Funding acquisition; Investigation; Methodology; Validation; Writing—original draft; Writing—review & editing.

## Supplementary Material

qsae024_Supplementary_Data

## Data Availability

All data and code to reproduce all results and visualizations reported herein have been made publicly available through online repositories (37).

## References

[1] Baker T.J. , Tyler C.R., Galloway T.S. (2014) Environ. Pollut. 186, 257–271. doi:10.1016/j.envpol.2013.11.014 24359692

[2] Hochella M.F. , Mogk D.W., Ranville J., Allen I.C., Luther G.W., Marr L.C., McGrail B.P., Murayama M., Qafoku N.P., Rosso K.M., Sahai N., Schroeder P.A., Vikesland P., Westerhoff P., Yang Y. (2019) Science 363, eaau8299. doi:10.1126/science.aau8299 30923195

[3] Montaño M.D. , von der Kammer F., Cuss C.W., Ranville J.F. (2019) J. Anal. At. Spectrom. 34, 1768–1772. doi:10.1039/c9ja00168a

[4] Canesi L. , Corsi I. (2016) Sci. Total Environ. 565, 933–940. doi:10.1016/j.scitotenv.2016.01.085 26805446

[5] Beyer J. , Green N.W., Brooks S., Allan I.J., Ruus A., Gomes T., Bråte I.L.N., Schøyen M. (2017) Mar. Environ. Res. 130, 338–365. doi:10.1016/j.marenvres.2017.07.024 28802590

[6] Ward J.E. , Kach D.J. (2009) Mar. Environ. Res. 68, 137–142. doi:10.1016/j.marenvres.2009.05.002 19525006

[7] FAO (2023) International markets for fisheries and aquaculture products – Fourth issue 2023, with January–June 2023 statistics. GLOBEFISH Highlights, No. 4–2023. Rome. 10.4060/cc9176en

[8] Mozhayeva D. , Engelhard C. (2020) J. Anal. At. Spectrom. 35, 1740–1783. doi:10.1039/c9ja00206e

[9] Laborda F. , Bolea E., Cepriá G., Gómez M.T., Jiménez M.S., Pérez-Arantegui J., Castillo J.R. (2016) Anal. Chim. Acta 904, 10–32. doi:10.1016/j.aca.2015.11.008 26724760

[10] Giusti A. , Atluri R., Tsekovska R., Gajewicz A., Apostolova M.D., Battistelli C.L., Bleeker E.A.J., Bossa C., Bouillard J., Dusinska M., Gómez-Fernández P., Grafström R., Gromelski M., Handzhiyski Y., Jacobsen N.R., Jantunen P., Jensen K.A., Mech A., Navas J.M., Nymark P., Oomen A.G., Puzyn T., Rasmussen K., Riebeling C., Rodriguez-Llopis I., Sabella S., Sintes J.R., Suarez-Merino B., Tanasescu S., Wallin H., Haase A. (2019) NanoImpact 16, 100182. doi:10.1016/j.impact.2019.100182

[11] Gao X. , Lowry G.V. (2018) NanoImpact 9, 14–30. doi:10.1016/j.impact.2017.09.002

[12] Laycock A. , Clark N.J., Clough R., Smith R., Handy R.D. (2022) Environ. Sci. Nano 9, 420–453. doi:10.1039/d1en00680k 35309016 PMC8852815

[13] Correia M. , Verleysen E., Loeschner K. (2019) in Nanomaterials for Food Applications, A.L. Rubio (Ed.), Elsevier, Amsterdam, Netherlands, pp 273–311. doi:10.1016/B978-0-12-814130-4.00010-5

[14] Sun Y. , Yang Y., Tou F.-Y., Niu Z.-S., Guo X.-P., Liu C., Yan J., Wu J.-Y., Xu M., Hou L.-J., Liu M. (2022) J. Hazard. Mater. 424, 127383. doi:10.1016/j.jhazmat.2021.127383 34879574

[15] Suzuki Y. , Harimoto M., Takahashi M., Akiyama H., Hirose A., Tsutsumi T. (2024) Kankyo. Kagaku. 34, 9–20doi:10.5985/jec.34.9

[16] Vidmar J. , Buerki-Thurnherr T., Loeschner K. (2018) J. Anal. At. Spectrom.33, 752–761. doi:10.1039/C7JA00402H

[17] Nguyen H.T.M. , Sylla K.S.B., Randriamahatody Z., Donnay-Moreno C., Moreau J., Tran L.T., Bergé J.P. (2011) *Food Technol. Biotechnol*. **49**, 48–55

[18] Liaset B. , Nortvedt R., Lied E., Espe M. (2002) Process Biochem. 37, 1263–1269. doi:10.1016/s0032-9592(02)00003-1

[19] Loeschner K. , Navratilova J., Købler C., Mølhave K., Wagner S., Von Der Kammer F., Larsen E.H. (2013) Anal. Bioanal. Chem. 405, 8185–8195. doi:10.1007/s00216-013-7228-z 23887279

[20] EC 625/2017 (2017) Off. J. Eur. Union L95, 1–142

[21] FAO (2010) International Conference on Food and Agriculture, FAO, Sao Pedro, Brazil

[22] Watkins P.S. , Castellon B.T., Tseng C., Wright M.V., Matson C.W., Cobb G.P. (2018) Bull. Environ. Contam. Toxicol. 100, 809–814. doi:10.1007/s00128-018-2336-2 29654375

[23] Sandrine Millour Y.N. (2015) J. Nanomed. Nanotechnol. 06, 1–8. doi:10.4172/2157-7439.1000269

[24] Witzler M. , Küllmer F., Günther K. (2018) Anal. Lett. 51, 587–599. doi:10.1080/00032719.2017.1327538

[25] Gray E.P. , Coleman J.G., Bednar A.J., Kennedy A.J., Ranville J.F., Higgins C.P. (2013) Environ. Sci. Technol. 47, 14315–14323. doi:10.1021/es403558c 24218983

[26] Loeschner K. , Navratilova J., Grombe R., Linsinger T.P.J., Købler C., Mølhave K., Larsen E.H. (2015) Food Chem. 181, 78–84. doi:10.1016/j.foodchem.2015.02.033 25794724

[27] Peters R.J.B. , Rivera Z.H., van Bemmel G., Marvin H.J.P., Weigel S., Bouwmeester H. (2014) Anal. Bioanal. Chem. 406, 3875–3885. doi:10.1007/s00216-013-7571-0 24390462

[28] Waegeneers N. , De Vos S., Verleysen E., Ruttens A., Mast J. (2019) Materials 12, 2677. doi:10.3390/ma12172677 31443380 PMC6747558

[29] Taboada-López M.V. , Herbello-Hermelo P., Domínguez-González R., Bermejo-Barrera P., Moreda-Piñeiro A. (2019) Talanta 195, 23–32. doi:10.1016/j.talanta.2018.11.023 30625537

[30] Linsinger T.P.J. , Chaudhry Q., Dehalu V., Delahaut P., Dudkiewicz A., Grombe R., von der Kammer F., Larsen E.H., Legros S., Loeschner K., Peters R., Ramsch R., Roebben G., Tiede K., Weigel S. (2013) Food Chem. 138, 1959–1966. doi:10.1016/j.foodchem.2012.11.074 23411331

[31] Gilroy K.D. , Neretina S., Sanders R.W. (2014) J. Nanopart. Res. 8, 1–8. doi:10.1016/gr5fgj

[32] von der Kammer F. , Ferguson P.L., Holden P.A., Masion A., Rogers K.R., Klaine S.J., Koelmans A.A., Horne N., Unrine J.M. (2012) Environ. Toxicol. Chem. 31, 32–49 doi:10.1002/etc723 22021021

[33] FAO and WHO (2019) Codex Alimentarius Commission—Procedural Manual, 27th Ed., Secretariat of the Joint FAO/WHO Food Standards Programme, Rome, Italy

[34] Duinker A. , Storesund J., Lundestad B.T., Sanden M. (2020) National Monitoring Program for Bivalves and Other Molluscs, Institute of Marine Research, Bergen, Norway

[35] Peters R. , Elbers I., Undas A., Sijtsma E., Briffa S., Carnell-Morris P., Siupa A., Yoon T.-H., Burr L., Schmid D., Tentschert J., Hachenberger Y., Jungnickel H., Luch A., Meier F., Kocic J., Kim J., Park B.C., Hardy B., Johnston C., Jurkschat K., Radnik J., Hodoroaba V.-D., Lynch I., Valsami-Jones E. (2021) Molecules 27, 5315. doi:10.3390/molecules27154849 PMC8433974 34500752

[36] Pace H.E. , Rogers N.J., Jarolimek C., Coleman V.A., Higgins C.P., Ranville J.F. (2011) Anal. Chem. 83, 9361–9369. doi:10.1021/ac201952t 22074486 PMC3410750

[37] Bruvold A. , Valdersnes S., Loeschner K., Solli B., Bienfait A.M. (2023) Open Source Repository for raw data: Validation of a method for surveillance of nanoparticles in mussels using single particle inductively coupled plasma mass spectrometry, Zenodo. doi:10.5281/zenodo.8301248 PMC11223760 38507699

[38] Julshamn K. , Lea P., Lindeberg J. (2009) Validation of Chemical Analytical Methods, Nordic Committee on Food Analysis, Bergen, Norway

[39] Magnusson B. , Örnemark U. (2014) Eurachem Guide: The Fitness for Purpose of Analytical Methods—A Laboratory Guide to Method Validation and Related Topics, 2nd Ed, EURACHEM, www.eurachem.org

[40] Ellison S.L.R. (2017) metRology: Support for Metrological Applications v0.9-28-1, https://cran.rproject.org/package=metRology (accessed March 30, 2023)

[41] May T.W. , Wiedmeyer R.H. (1998) At. Spectrosc. 19, 150–155

[42] Kálomista I. , Kéri A., Ungor D., Csapó E., Dékány I., Prohaska T., Galbács G. (2017) J. Anal. At. Spectrom. 32, 2455–2462. doi:10.1039/c7ja00306d

[43] Gonzalez de Vega R. , Lockwood T.E., Xu X., Gonzalez de Vega C., Scholz J., Horstmann M., Doble P.A., Clases D. (2022) Anal. Bioanal. Chem. 414, 5671–5681. doi:10.1007/s00216-022-04052-0 35482065 PMC9242955

[44] Torregrosa D. , Gómez-Pertusa C., Grindlay G., Gras L., Mora J. (2023) J. Anal. At. Spectrom.38, 403–413. doi:10.1039/D2JA00342B

[45] Liu J. , Murphy K.E., Winchester M.R., Hackley V.A. (2017) Anal. Bioanal. Chem. 409, 6027–6039. doi:10.1007/s00216-017-0530-4 28815280 PMC5693768

[46] Bruvold A.S. , Bienfait A.M., Ervik T.K., Loeschner K., Valdersnes S. (2023) Mar. Environ. Res. 188, 105975. doi:10.1016/j.marenvres.2023.105975 37086530

[47] Witzler M. , Küllmer F., Hirtz A., Günther K. (2016) J. Agric. Food Chem. 64, 4165–4170. doi:10.1016/f8p9k9 27132879

[48] Tuoriniemi J. , Cornelis G., Hassellöv M. (2014) J. Anal. Spectrom. 29, 743–752. doi:10.1039/C3JA50367D

[49] Stewart I.I. , Olesik J.W. (1999) J. Am. Soc. Mass Spectrom. 10, 159–174. doi:10.1016/S1044-0305(98)00136-6 9926408

[50] Torregrosa D. , Grindlay G., Gras L., Mora J. (2023) J. Anal. At. Spectrom. 38, 1874–1884. doi:10.1039/D3JA00134B

[51] Laborda F. , Bolea E., Jiménez-Lamana J. (2016) Anal. Chem. 9, 15–23. doi:10.1016/j.teac.2016.02.001 24308527

[52] Schardt A. , Schmitt J., Engelhard C. (2022) *ChemRxiv* preprint. doi:10.26434/chemrxiv-2022-rc0c2

[53] Ho K.-S. , Lui K.-O., Lee K.-H., Chan W.-T. (2013) Spectrochim. Acta Part B At. Spectrosc. 89, 30–39. doi:10.1016/j.sab.2013.08.012

[54] Olesik J.W. , Gray P.J. (2012) J. Anal. At. Spectrom. 27, 1143–1155. doi:10.1039/c2ja30073g

[55] Lee W.-W. , Chan W.-T. (2015) J. Anal. At. Spectrom. 30, 1245–1254. doi:10.1039/C4JA00408F

[56] EC C 229/01/2022 (2022) Off. J. Eur. Union C229, 1–33

[57] Olesik J.W. (2014) in Treatise on Geochemistry, Elsevier, pp 309–336. doi:10.1016/B978-0-08-095975-7.01426-1

[58] Gomez-Gomez B. , Perez-Corona M.T., Madrid Y. (2020) Anal. Chim. Acta 1100, 12–21. doi:10.1016/j.aca.2019.11.063 31987132

[59] Geiss O. , Bianchi I., Senaldi C., Bucher G., Verleysen E., Waegeneers N., Brassinne F., Mast J., Loeschner K., Vidmar J., Aureli F., Cubadda F., Raggi A., Iacoponi F., Peters R., Undas A., Müller A., Meinhardt A.-K., Walz E., Gräf V., Barrero-Moreno J. (2021) Food Control. 120, 107550. doi:10.1016/j.foodcont.2020.107550 33536722 PMC7730118

[60] Jiménez-Lamana J. , Marigliano L., Allouche J., Grassl B., Szpunar J., Reynaud S. (2020) Anal. Chem.92, 11664–11672. doi:10.1021/acs.analchem.0c01536 32786493

[61] Taboada-López M.V. , Alonso-Seijo N., Herbello-Hermelo P., Bermejo-Barrera P., Moreda-Piñeiro A. (2019) Microchem. J. 148, 652–660. doi:10.1016/j.microc.2019.05.023

[62] Taboada-López M.V. , Iglesias-López S., Herbello-Hermelo P., Bermejo-Barrera P., Moreda-Piñeiro A. (2018) Anal. Chim. Acta. 1018, 16–25. doi:10.1016/j.aca.2018.02.075 29605130

[63] Zhou Q. , Liu L., Liu N., He B., Hu L., Wang L. (2020) Ecotoxicol. Environ. Saf. 198, 110670. doi:10.1016/j.ecoenv.2020.110670 32344268

[64] Mech A. , Wohlleben W., Ghanem A., Hodoroaba V., Weigel S., Babick F., Brüngel R., Friedrich C.M., Rasmussen K., Rauscher H. (2020) Small 16, e2002228. doi:10.1002/smll.202002228 32743899

[65] Weigel S. , Peters R., Loeschner K., Grombe R., Linsinger T.P.J. (2017) Anal. Bioanal. Chem. 409, 4839–4848. doi:10.1007/s00216-017-0427-2 28634763 PMC5519662

[66] Salou S. , Larivière D., Cirtiu C.-M., Fleury N. (2021) Anal. Bioanal. Chem. 413, 171–181. doi:10.1007/s00216-020-02989-8 33123763

[67] De La Calle I. , Menta M., Klein M., Séby F. (2018) Food Chem. 266, 133–145. doi:10.1016/j.foodchem.2018.05.107 30381168

[68] Montoro Bustos A.R. , Murphy K.E., Winchester M.R. (2022) Anal. Chem. 94, 3091–3102. doi:10.1021/acs.analchem.1c04140 35144383 PMC9809148

[69] Malysheva A. , Ivask A., Hager C., Brunetti G., Marzouk E.R., Lombi E., Voelcker N.H. (2016) Nanotoxicology 10, 385–390. doi:10.3109/17435390.2015.1084059 26472210

[70] Suzuki Y. , Kondo M., Akiyama H., Ogra Y. (2022) Environ. Pollut. 307, 119555. doi:10.1016/j.envpol.2022.119555 35654251

[71] Xu L. , Wang Z., Zhao J., Lin M., Xing B. (2020) Environ. Pollut. 260, 114043. doi:10.1016/j.envpol.2020.114043 32041024

[72] Wilkinson K.J. , Lead J.R. (2007) Environmental Colloids and Particles: Behaviour, Separation and Characterisation, John Wiley & Sons, Ltd, Chichester, England

