# Supplementary material for: Validation of a Method for Surveillance of Nanoparticles in Mussels Using Single-Particle Inductively Coupled Plasma-Mass Spectrometry
Source: J AOAC Int. 2024 Mar 20;107(4):608–16. doi: 10.1093/jaoacint/qsae024 (PMC11223760; doi:10.1093/jaoacint/qsae024)
Supplement: qsae024_Supplementary_Data [file qsae024_supplementary_data.docx]

# Supplementary

Supplementary Table 1: Mean particle diameter

| Sample/ Replicate | Day | | | | |
| --- | --- | --- | --- | --- | --- |
|  | 1 | 2 | 3 | 4 | 5 |
| Method blank + 60 nm | | | | | |
| 1 | 48.10 | 51.37 | 51.21 | 56.21 | 54.71 |
| 2 | 35.70 | 53.11 | 52.06 | 52.84 | 53.81 |
| Method blank + 30 nm | | | | | |
| 1 | 32.32 | 33.02 | 33.39 | 33.18 | 33.43 |
| 2 | 32.75 | 32.82 | 33.20 | 33.44 | 33.56 |
| Matrix blank + 60 nm | | | | | |
| 1 | 54.07 | 52.76 | 53.64 | 55.56 | 54.93 |
| Matrix blank + 30 nm | | | | | |
| 1 | 32.48 | 32.73 | 32.96 | 33.07 | 32.95 |
| Instrument blank + 60 nm | | | | | |
| 1 | 59.47 | 58.31 | 57.84 | 60.00 | 59.90 |
| Instrument blank + 30 nm | | | | | |
| 1 | 35.52 | 35.67 | 35.74 | 35.68 | 35.76 |
| Blue mussel + 60 nm | | | | | |
| 1 | 52.92 | 53.13 | 53.34 | 54.23 | 52.32 |
| 2 | 52.45 | 53.01 | 53.00 | 54.59 | 54.01 |
| Blue mussel + 30 nm | | | | | |
| 1 | 32.54 | 32.86 | 34.19 | 33.31 | 33.13 |
| 2 | 32.46 | 32.50 | 33.15 | 32.88 | 32.93 |
| Blue mussel + 30 and 60 nm | | | | | |
| 1 | 34.93 | 35.25 | 36.19 | 35.63 | 36.14 |
| 2 | 35.10 | 35.46 | 36.16 | 35.56 | 36.13 |

Supplementary Table 2: Particle mass concentration [ng/kg mussel tissue]

| Sample/ Replicate | Day | | | | |
| --- | --- | --- | --- | --- | --- |
|  | 1 | 2 | 3 | 4 | 5 |
| Method blank + 60 nm | | | | | |
| 1 | 1.22 | 3.43 | 3.63 | 3.99 | 3.81 |
| 2 | 1.35 | 3.15 | 2.79 | 2.52 | 3.35 |
| Method blank + 30 nm | | | | | |
| 1 | 4.38 | 5.34 | 4.88 | 6.13 | 4.66 |
| 2 | 10.21 | 5.13 | 5.34 | 5.54 | 5.31 |
| Matrix blank + 60 nm | | | | | |
| 1 | 20.99 | 18.93 | 19.35 | 18.62 | 15.56 |
| Matrix blank + 30 nm | | | | | |
| 1 | 33.02 | 30.18 | 27.21 | 26.50 | 26.87 |
| Instrument blank + 60 nm | | | | | |
| 1 | 21.49 | 20.62 | 24.81 | 25.31 | 23.57 |
| Instrument blank + 30 nm | | | | | |
| 1 | 35.06 | 33.28 | 34.88 | 35.89 | 32.74 |
| Blue mussel + 60 nm | | | | | |
| 1 | 19.85 | 17.23 | 18.01 | 15.25 | 16.52 |
| 2 | 18.18 | 17.93 | 19.86 | 16.20 | 17.02 |
| Blue mussel + 30 nm | | | | | |
| 1 | 28.99 | 26.08 | 27.60 | 27.06 | 24.18 |
| 2 | 30.01 | 25.09 | 25.68 | 25.60 | 23.99 |
| Blue mussel + 30 and 60 nm | | | | | |
| 1 | 44.40 | 45.53 | 43.06 | 40.72 | 38.49 |
| 2 | 45.12 | 44.59 | 41.84 | 44.12 | 39.44 |

Supplementary Table 3: Particle number concentration [ng/kg mussel tissue]

| Sample/ Replicate | Day | | | | |
| --- | --- | --- | --- | --- | --- |
|  | 1 | 2 | 3 | 4 | 5 |
| Method blank + 60 nm | | | | | |
| 1 | 8.92 x 10^5 | 2.21 x 10^6 | 2.21 x 10^6 | 2.02 x 10^6 | 2.04 x 10^6 |
| 2 | 1.84 x 10^6 | 1.85 x 10^6 | 1.71 x 10^6 | 1.46 x 10^6 | 1.91 x 10^6 |
| Method blank + 30 nm | | | | | |
| 1 | 1.16 x 10^7 | 1.29 x 10^7 | 1.20 x 10^7 | 1.55 x 10^7 | 1.14 x 10^7 |
| 2 | 2.53 x 10^7 | 1.33 x 10^7 | 1.26 x 10^7 | 1.36 x 10^7 | 1.29 x 10^7 |
| Matrix blank + 60 nm | | | | | |
| 1 | 1.13 x 10^7 | 1.14 x 10^7 | 1.09 x 10^7 | 1.01 x 10^7 | 8.52 x 10^6 |
| Matrix blank + 30 nm | | | | | |
| 1 | 8.45 x 10^7 | 7.63 x 10^7 | 6.85 x 10^7 | 6.88 x 10^7 | 6.96 x 10^7 |
| Instrument blank + 60 nm | | | | | |
| 1 | 9.59 x 10^6 | 9.43 x 10^6 | 1.11 x 10^7 | 1.07 x 10^7 | 1.01 x 10^7 |
| Instrument blank + 30 nm | | | | | |
| 1 | 7.35 x 10^7 | 6.90 x 10^7 | 7.21 x 10^7 | 7.43 x 10^7 | 6.71 x 10^7 |
| Blue mussel + 60 nm | | | | | |
| 1 | 1.13 x 10^7 | 1.04 x 10^7 | 1.06 x 10^7 | 8.88 x 10^6 | 9.99 x 10^6 |
| 2 | 1.05 x 10^7 | 1.09 x 10^7 | 1.10 x 10^7 | 9.29 x 10^6 | 9.80 x 10^6 |
| Blue mussel + 30 nm | | | | | |
| 1 | 7.58 x 10^7 | 6.72 x 10^7 | 6.23 x 10^7 | 6.85 x 10^7 | 6.17 x 10^7 |
| 2 | 7.24 x 10^7 | 6.84 x 10^7 | 6.47 x 10^7 | 6.76 x 10^7 | 6.26 x 10^7 |
| Blue mussel + 30 and 60 nm | | | | | |
| 1 | 8.36 x 10^7 | 8.04 x 10^7 | 7.20 x 10^7 | 7.33 x 10^7 | 6.67 x 10^7 |
| 2 | 8.45 x 10^7 | 8.01 x 10^7 | 7.14 x 10^7 | 7.90 x 10^7 | 6.77 x 10^7 |

Table 4: Response factors and linearity of the calibration curve across all days based on four Au ionic standards from 0 - 10 μg/L.

| Day | Intercept [counts/dwell] | Response [counts/dwell] | R^2^ |
| --- | --- | --- | --- |
| 1 | 0.02 | 6.45 | 0.99999 |
| 2 | -0.06 | 6.81 | 0.99999 |
| 3 | -0.14 | 7.15 | 0.99998 |
| 4 | -0.10 | 7.14 | 0.99999 |
| 5 | -0.20 | 8.24 | 0.99997 |

### Trueness

Equation 1 $Recovery (\%)= \frac{Measured value in matrix}{Measured value in UPW}\cdot100\%$

Equation 2 $z-score= \frac{Measured value-Concensus value from PT}{Standard deviation of the PT}$

### Measurement uncertainty

To determine the signal per element mass, $S$, a particulate reference material with a known mass or size can be analyzed. By integrating background-subtracted peaks and using the median or the kernel density estimate of the resulting signal distribution, we obtain an estimate of the signal per particle mass or size in [counts/kg]:

$$S=\frac{6A_{rm}}{\rho\cdot\pi\cdot d_{rm}^{3}},$$

where $\rho$ is the element density, $d_{rm}$ the particle diameter and $A_{rm}$ is the estimated signal per particle in counts.

Using ionic standards to establish a calibration curve, we use the slope coefficient, $b$, and the concentration of the corresponding ionic standard, $C_{std}$, for an estimate for the signal per dwell per mass element per volume in $\frac{counts}{dwell}/\frac{kg}{L}$:

$$I=\frac{b}{C_{std}}.$$

Combining these and multiplying by $t_{meas}$, the number of dwells during acquisition, the flow rate through the plasma, $Q_{det}$, in $\frac{L}{t_{acq}}$ may be determined and used to relate measured quantities to concentrations:

$$Q_{det}=\frac{t_{meas}\cdot I}{S}.$$

Mean particle diameter is then given by

$$\overline{d}_{p}=\frac{1}{n}\sum_{i=1}^{n} d_{p_{i}}=\frac{1}{n}\sum_{i=1}^{n} \sqrt[3]{\frac{6\cdot A_{p_{i}}}{S\pi\rho}}=\frac{1}{n}\sum_{i=1}^{n} \sqrt[3]{\frac{A_{p_{i}}\cdot\rho_{rm}\cdot d_{rm}^{3}}{A_{rm}\cdot\rho}}$$

the particle mass concentration, $c_{m}$, by

$$c_{m}=\frac{\sum_{i=1}^{n} A_{p_{i}}}{{S\cdot t}_{meas}\cdot Q_{det}}=\frac{C_{std}\sum_{i=1}^{n} A_{p_{i}}}{t_{meas}\cdot b},$$

and the particle number concentration, $c_{n}$, by

$$c_{n}=\frac{n_{meas}}{{t_{meas}\cdot Q}_{det}}.$$

Inserting for $Q_{det}$, we get

$$c_{n}=\frac{n_{meas}\cdot6A_{rm}\cdot C_{std}}{t_{meas}\cdot\rho\cdot\pi\cdot d_{rm}^{3}\cdot b}$$

- $n_{meas}$ is the instrument measured number of particle events. This corresponds to a Poisson process and hence the standard uncertainty will be its square root. Using the LOD for particle number concentration based on 35 measured particles justifies a normal approximation as a conservative estimate for the uncertainty.
- $A_{rm}$ [counts] is the location of the peak area per particle event of the reference material as determined using a kernel density estimate, uncertainty set to **5%**.
- $A_{p}$ [counts] is the peak area or total signal of a particle event of which **5 %** uncertainty is assumed.
- $C_{std}$ [kg/L] is the concentration of the standard. We use the uncertainty of the standard at **1%,** and add another multiplicative term with **5%** to account for the uncertainty of preparation.
- $t_{meas}$ is the number of dwells per measurement, uncertainty set to 0.5%.
- $\rho$ [kg/m^3^] is the element density of a particle. Its uncertainty is not well-established, **0.05%** having been used [1]. We set a more conservative value at **1%**.
- $d$ [m] is the particle diameter. For the reference material, the uncertainty in the certificate of analysis at **4 %** is used.
- $b$ [counts/dwell] is the slope coefficient of the calibration curve. We use the uncertainty of the the linear regression for each day.

For each variable the standard uncertainty is assumed for a Monte Carlo simulation with ${10}^{6}$ repeats, reporting the uncertainty with a coverage factor of two. Note that for particles of other elements than gold, the additional uncertainty for calculating from using another ionic standard applies:

$$S_{i}=S\frac{C_{std}\cdot b_{i}}{C_{i}\cdot b}$$

where $S_{i}$ is the signal per particle mass for element $i$, $C_{i}$ is the concentration of the ionic standard for element $i$ and $b_{i}$ is the slope coefficient of the calibration curve for element $i$.

Supplementary Table 5: Recoveries of particle concentration, mass concentration and particle diameters in different sample types in comparison to nominal values of the 30 and 60 nm NP reference materials, PerkinElmer 60 nm Spherical Gold (Au) Nanoparticle in DI Water 25 mL (N8142303) and PerkinElmer 30 nm Spherical Gold (Au) Nanoparticle in DI Water 25 ml (N8142300), nominal gold mass concentrations 50 mg/L, calculated number concentrations based on mean diameters 30 and 60.

| Sample | Particle diameter | Particle mass concentration | Particle number concentration |
| --- | --- | --- | --- |
| 30 nm gold NPs spiked to | | | |
| Blue mussel | 110% | 53% | 37% |
| BM spike after enzymatic digestion/Matrix blank | 109% | 57% | 40% |
| Method blank | 110% | 11% | 8% |
| UPW/Instrument blank | 119% | 69% | 39% |
| 60 nm gold NPs spiked to | | | |
| Blue mussel | 89% | 35% | 45% |
| BM spike after enzymatic digestion/Matrix blank | 90% | 37% | 46% |
| Method blank | 85% | 6% | 8% |
| Instrument blank (UPW) | 99% | 46% | 44% |

*Table 6: Instrumental parameters used for the validation study.*

| Parameter | Value |
| --- | --- |
| RF power | 1550 W |
| Sampling depth | 8.0 mm |
| Nebulizer gas flow | 1.05 L/min |
| Nebulizer | Concentric **(**MicroMist) |
| Peristaltic pump speed | 0.1 rps |
| Torch (inner diameter of injector) | 1.5 mm |
| Tubing inner diameter | 1.02 mm |
| Sample inlet flow | 0.35 mL/min |
| Integration (dwell) time | 100 µs (peak integration mode) |
| Isotope monitored | ^197^Au |
| Analysis time | 120 s |
| Probe rinse | Rinse 1: 60 s 5% v/v concentrated HNO_3_  Rinse 2: 90 s UPW |

*Supplementary Table 7: Instrumental parameters used for the method applicability demonstration.*

| Parameter | Value |
| --- | --- |
| RF power | 1550 W |
| Sampling depth | 8.0 mm |
| Nebulizer gas flow | 1.05 L/min |
| Nebulizer | Concentric **(**MicroMist) |
| Peristaltic pump speed | 0.1 rps |
| Torch (inner diameter of injector) | 1.5 mm |
| Tubing inner diameter | 1.02 mm |
| Sample inlet flow | 0.35 mL/min |
| Integration (dwell) time | 100 µs (peak integration mode) |
| Isotope monitored | ^197^Au, ^48^Ti, ^52^Cr, ^63^Cu |
| Analysis time | 180 s |
| Probe rinse (0.5 RPS) | Rinse 1: 60 s 1% HNO_3_/HCl/Triton-X  Rinse 2: 30 s 5% v/v concentrated HNO_3_  Rinse 2: 60 s UPW |

Supplementary Table 8: Particle diameters for method applicability demonstration

|  | **Mean [nm]** | **Detection limit [nm]** | **RSD_Repeatability_** | **RSD_Intermediate precision_** |
| --- | --- | --- | --- | --- |
| Ti | | | | |
| Farm | 67 | 45 | 2% | *^*^*2% |
| Harbor | 64 | 45 | 3% | *^*^*3% |
| Surveillance | 59 | 45 | 4% | 6% |
| Cr | | | | |
| Farm | 49 | 44 | 3% | *^*^*3% |
| Harbor | 49 | 44 | 3% | *^*^*3% |
| Surveillance | 43 | 44 | 3% | 5% |
| Cu | | | | |
| Farm | 42 | 36 | 3% | *^*^*2% |
| Harbor | 48 | 36 | 1% | 2% |
| Surveillance | 42 | 36 | 1% | 2% |
| *^*^*RSD is presented as variance was lower between days than within days. | | | | |

Supplementary Table 9: Particle number concentrations for method applicability demonstration

|  | **Mean [#/g]** | **Detection limit [#/g]** | **RSD _Repeatability_** | **RSD_Intermediate precision_** |
| --- | --- | --- | --- | --- |
| Ti | | | | |
| Farm | 5.6 × 10^7 | 1.6 × 10^6 | 5% | *^*^*5% |
| Harbor | 5.4 × 10^7 | 1.6 × 10^6 | 7% | *^*^*6% |
| Surveillance | 1.6 × 10^7 | 1.6 × 10^6 | 10% | *^*^*10% |
| Cr | | | | |
| Farm | 5.4 × 10^6 | 8.1 × 10^5 | 11% | 11% |
| Harbor | 1.6 × 10^7 | 8.1 × 10^5 | 9% | 10% |
| Surveillance | 2.9 × 10^6 | 8.1 × 10^5 | 13% | 21% |
| Cu | | | | |
| Farm | 6.1 × 10^7 | 2.0 × 10^6 | 12% | *^*^*11% |
| Harbor | 6.3 × 10^7 | 2.0 × 10^6 | 18% | 24% |
| Surveillance | 5.9 × 10^7 | 2.0 × 10^6 | 9% | 14% |
| *^*^*RSD is presented as variance was lower between days than within days. | | | | |

**Supplementary Table 10**: Quality control parameters of analyte concentrations detected in the procedural blank (n=5 per day) and the mass concentration recovery of TiO_2_ NPs (JRCNM10200a) spiked into mussels (n = 3 per day) prior to incubation versus freshly prepared  TiO_2_ in UPW (n = 1 per day).

| Day | Element | Mass concentration  [ng/g] | Particle number concentration  [#/g] | Mass concentration recovery |
| --- | --- | --- | --- | --- |
| 1 | Cr | 0.1 | 189,565 | - |
| 1 | Cu | 0.0 | 50,551 | - |
| 1 | Ti | 2.0 | 429,680 | 93% |
| 2 | Cr | 0.9 | 206,722 | - |
| 2 | Cu | 0.3 | 2,080,143 | - |
| 2 | Ti | 0.9 | 1,033,611 | 81% |

Supplementary figure 1: Particle number concentration in wet mussel tissue, error bars indicating the 95% CI of the mean, red line denoting the detection limit.


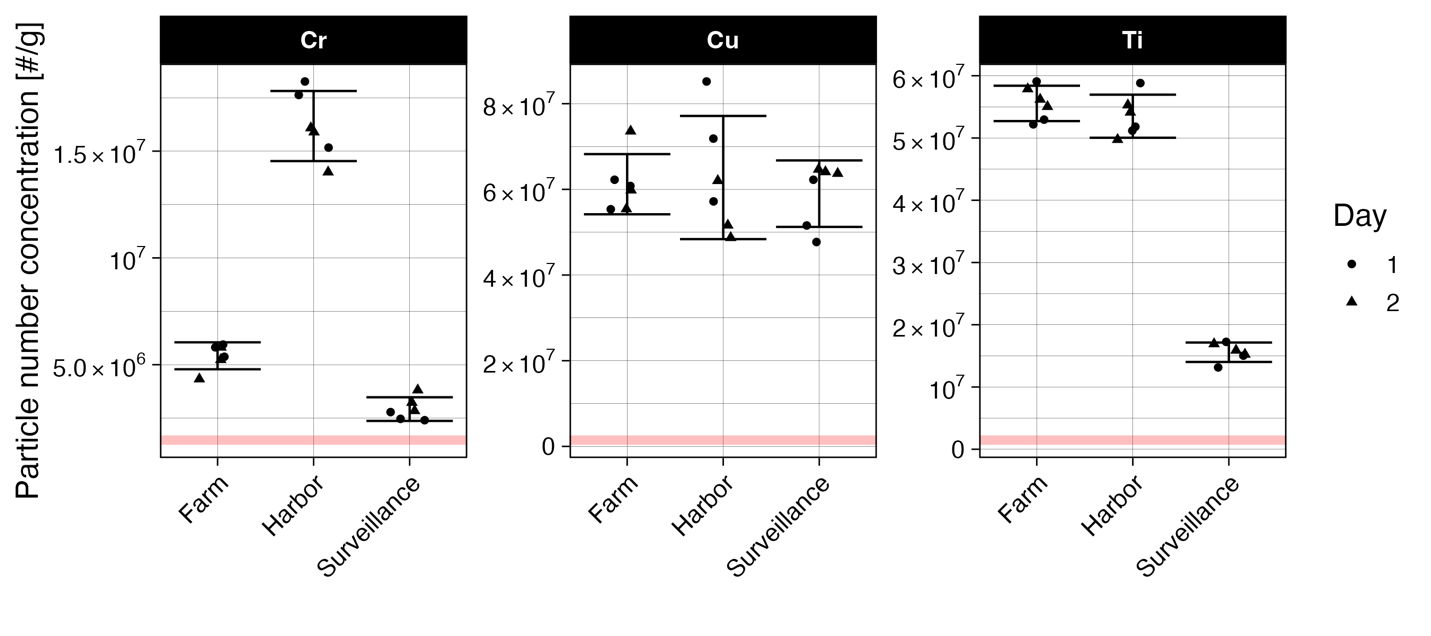


**
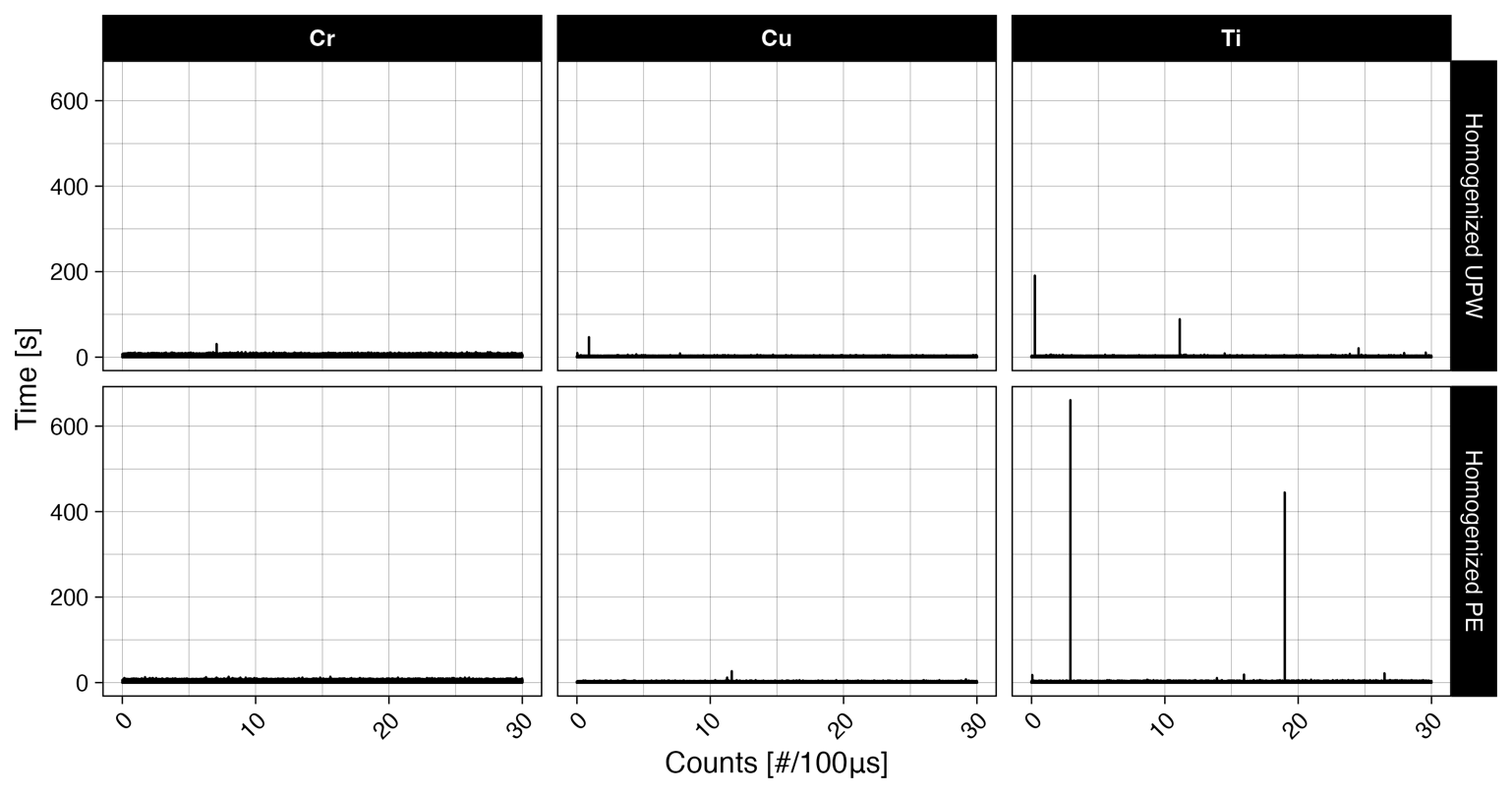
**

Supplementary figure 2: Time scans of UPW blanks having gone through the homogenization procedure. The top showing homogenized UPW, bottom showing homogenized UPW additionally containing polyethylene beads to simulate the mechanical abrasion exerted on the blades.


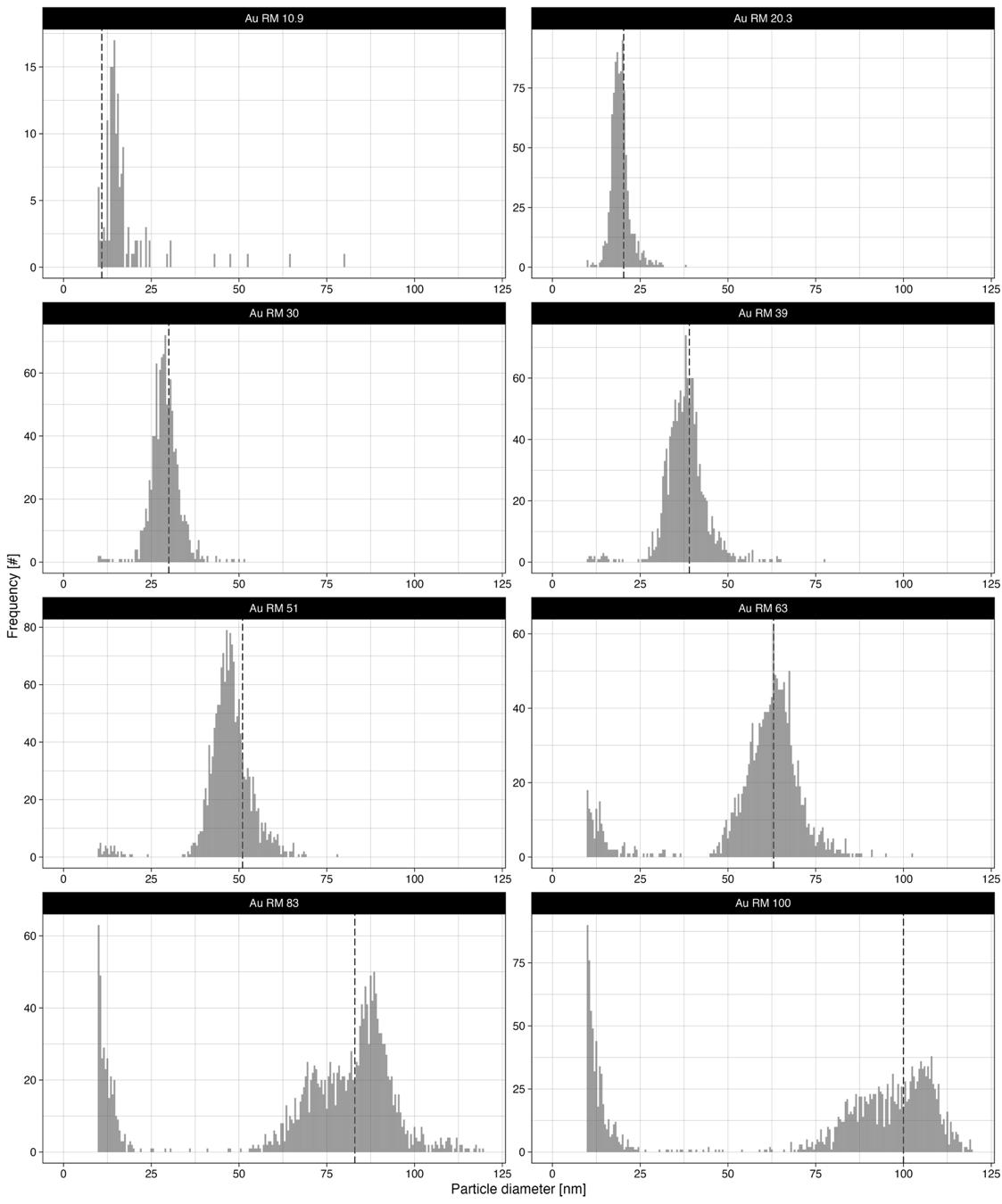


Supplementary figure 3: Different sizes of Au NPs (nanoComposix Gold Nanospheres, 50 mg/L) spiked into UPW analyzed with an Agilent 8900 using with similar setup and same sampling depth and nebulizer gas, illustrating the size-dependence of the artifact.


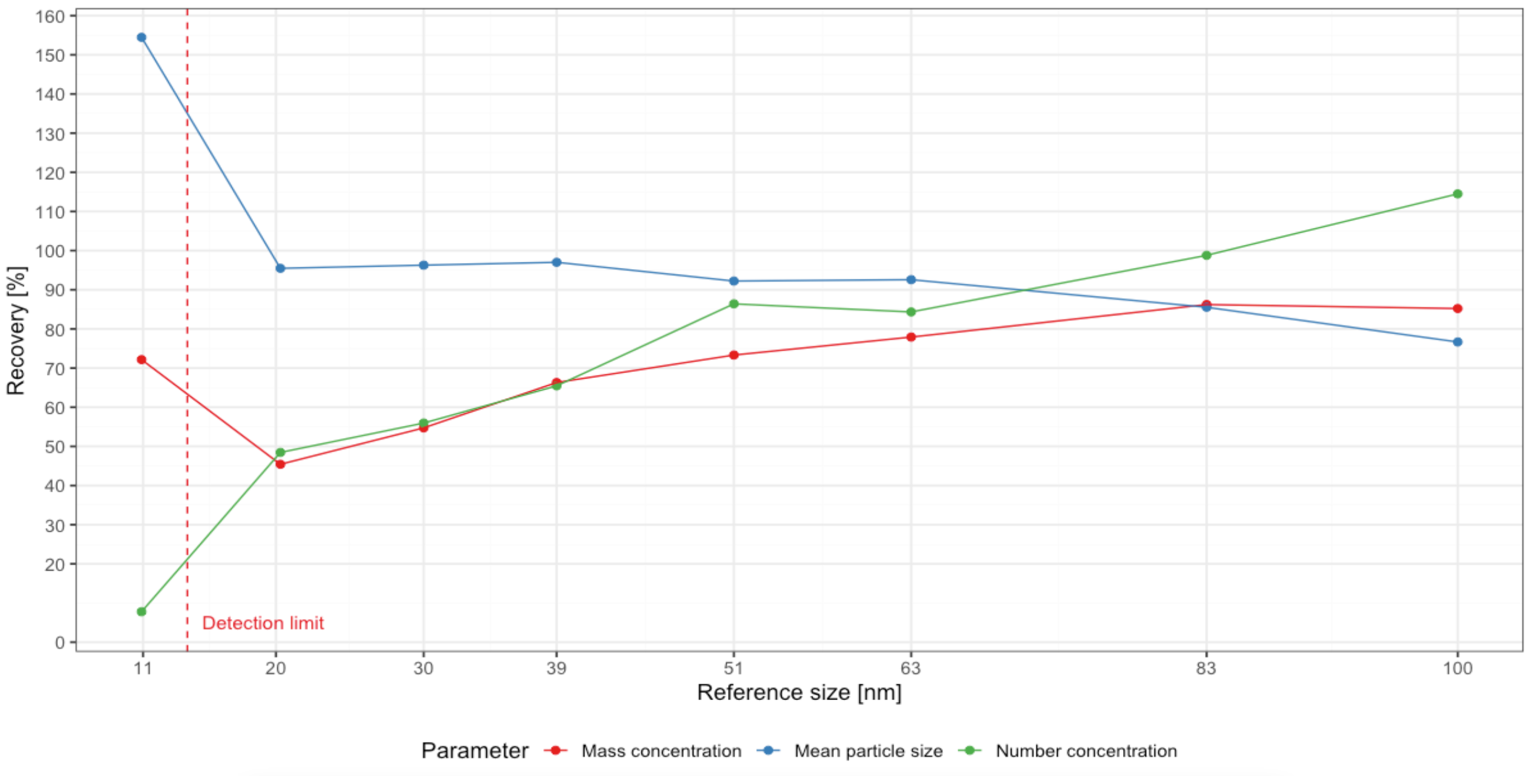


Supplementary figure 4: Gold NPs (nanoComposix NanoXact gold nanospheres 11 - 100 nm) at nominal concentrations increasing with size at 1, 7, 20, 50, 100, 200, 500 and 800 ng/L spiked into UPW, showing the recovery in dependence of particle size for the size, mass concentration and number concentration. Analyzed using an Agilent 8900 with similar setup as for the validation study, same sampling depth and nebulizer gas.

**Code**

The code used for data processing and statistical analysis is included in the GitHub repository https://github.com/arebruvold/mussel_validation/ .
